# Prediction of unconventional protein secretion by exosomes

**DOI:** 10.1186/s12859-021-04219-z

**Published:** 2021-06-16

**Authors:** Alvaro Ras-Carmona, Marta Gomez-Perosanz, Pedro A. Reche

**Affiliations:** Laboratory of Immunomedicine, Department of Immunology, Faculty of Medicine, Complutense University of Madrid, Pza Ramón y Cajal, s/n, 28040 Madrid, Spain

**Keywords:** Exosomes, Protein secretion, Random forests

## Abstract

**Motivation:**

In eukaryotes, proteins targeted for secretion contain a signal peptide, which allows them to proceed through the conventional ER/Golgi-dependent pathway. However, an important number of proteins lacking a signal peptide can be secreted through unconventional routes, including that mediated by exosomes. Currently, no method is available to predict protein secretion via exosomes.

**Results:**

Here, we first assembled a dataset including the sequences of 2992 proteins secreted by exosomes and 2961 proteins that are not secreted by exosomes. Subsequently, we trained different random forests models on feature vectors derived from the sequences in this dataset. In tenfold cross-validation, the best model was trained on dipeptide composition, reaching an accuracy of 69.88% ± 2.08 and an area under the curve (AUC) of 0.76 ± 0.03. In an independent dataset, this model reached an accuracy of 75.73% and an AUC of 0.840. After these results, we developed ExoPred, a web-based tool that uses random forests to predict protein secretion by exosomes.

**Conclusion:**

ExoPred is available for free public use at http://imath.med.ucm.es/exopred/. Datasets are available at http://imath.med.ucm.es/exopred/datasets/.

**Supplementary Information:**

The online version contains supplementary material available at 10.1186/s12859-021-04219-z.

## Background

Protein secretion is of paramount relevance for cellular communication [1]. In eukaryotes, most secreted proteins follow the classical endoplasmic reticulum (ER)-Golgi pathway. This pathway requires the presence of a signal peptide in the N-terminus of proteins (leader sequence), which promotes the delivery of nascent proteins into the lumen of the ER. Proteins are then transported to the Golgi apparatus and from there to the cellular surface via vesicular transport [2, 3].

In addition, there are unconventional pathways of protein secretion, which actually enable the secretion of leaderless proteins. Unconventional secretion of proteins can occur through vesicular and non-vesicular pathways [3, 4]. In non-vesicular pathways, proteins are released directly to the extracellular space, while in vesicular pathways, proteins are released within vesicles. Cells can secrete to the extracellular environment a variety of vesicular structure, among which exosomes stand out [5].

Exosomes are microvesicles ranging from 30 to 100 nm in size, playing an important role in intercellular communication thanks to their capacity to transport and transfer proteins, lipids and nucleic acids to other cells [6]. Exosome secretion has been involved in many biological processes, both in health and disease [7]. For example, exosomes are involved in the regulation of coagulation and inflammation [6, 8]. Interestingly, the content of exosomes can change under pathological conditions such as cancer, neurodegenerative ailments and cardiovascular diseases [7, 9, 10].

Exosomes are generated in the cytosol from inward budding invaginations of late endosomes, which results in intraluminal vesicles (ILV) within a large multivesicular body (MVB) [11]. When MVBs fuse with the plasma membrane, ILV are secreted into the extracellular space as exosomes [2, 12, 13]. Exosomes incorporate transmembrane proteins and a great variety of luminal cargo proteins, including cytosolic and nuclear proteins, lacking a signal peptide [14, 15].

Given the biological relevance of exosomes and their role in unconventional protein secretion, it is of great interest to identify and predict proteins secreted by these vesicles. Currently, there are several bioinformatics tools to predict proteins secreted through unconventional pathways [16], including SecretomeP [17], SPRED [18], SecretP [19, 20] and OutCyte [21]. All these tools are based on machine learning models that were not trained for the specific task of predicting protein secretion by exosomes. Here, we present ExoPred, a bioinformatic tool that is specific to predict proteins secreted by exosomes. ExoPred implements a Random Forests (RF) model that was trained on a sequence dataset including 2992 exosome luminal proteins assembled *ex profeso*. The dataset only included non-transmembrane and leaderless proteins from vertebrata. In tenfold cross-validation the model reached an accuracy of 69.88% ± 2.08 and an area under the curve (AUC) of 0.76 ± 0.03. Moreover, when tested in an independent dataset, this model reached an accuracy of 75.73%.

## Results and discussion

### Exosome training dataset

Proteins secreted by exosomes are really diverse with regard to structure, function and sub-cellular location [22, 23]. Here, we aimed to predict proteins secreted within exosomes: luminal cargo proteins. To that end, we generated a non-redundant dataset containing 2992 proteins found within exosomes and 2961 that are not found in exosomes. In the dataset, we only considered highly curated proteins from vertebrates with less than 80% identity, discarding membrane bound proteins or carrying a leader sequence, as they could be secreted by other means (details in Methods). The average sequence identity between exosome proteins included in the training dataset is 12.22 ± 2.02 (%), while the sequence identity between non-exosome proteins is 12.23 ± 2.38 (%). Overall, the average sequence identity in the training dataset is 12.19 ± 2.08 (%). In Additional file 1: Figure S1, we show additional measures of identity in the training dataset.

**Fig. 1:**
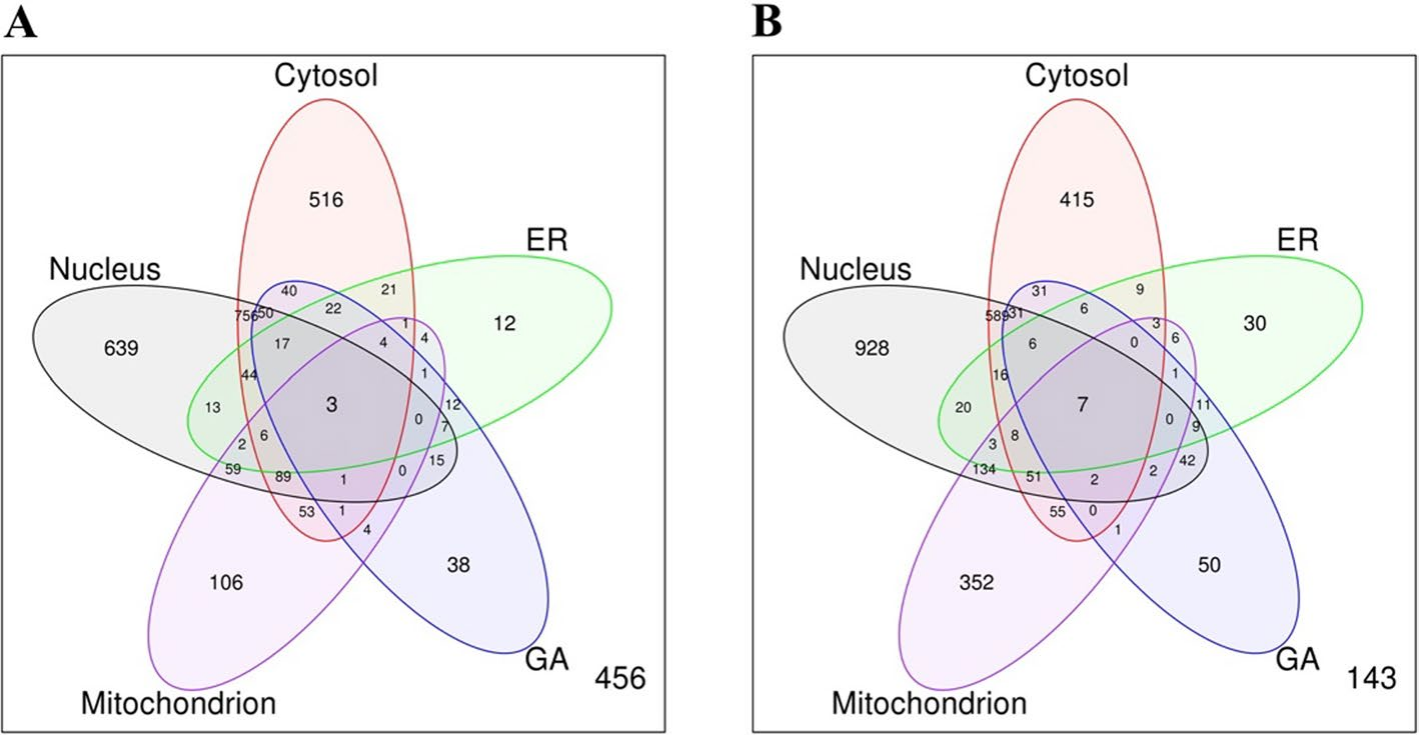
Sub-cellular location of proteins included in the training dataset. Venn diagrams indicating the sub-cellular location of exosome (**a**) and non-exosome proteins (**b**) as annotated in UNIPROT. Note that the preferential sub-cellular location for exosome and non-exosome proteins was cytosolic and nuclear

We investigated the sub-cellular location of exosome proteins upon UNIPROT annotations as a mean to select appropriated non-exosome proteins for inclusion in the training dataset (details in Methods). As shown in Fig. 1a, some exosome proteins have no sub-cellular location annotations (456) while many others (1225) have more than one sub-cellular location. The dataset also includes 639, 516, 12, 38 and 106 exosome proteins that are annotated with exclusive sub-cellular locations in the nucleus, cytosol, ER, GA and mitochondrion, respectively. Likewise, non-exosome proteins included in the training dataset exhibit sub-cellular locations that mirror those of exosome proteins (Fig. 1b). By including in the training dataset exosome and non-exosome proteins with balanced sub-cellular locations, we aimed to obtain prediction models that were robust and unbiased.

### Generation and evaluation of RF models predicting proteins secreted by exosomes

Luminal exosome proteins are very heterogeneous and, unlike proteins secreted by the classical secretion pathway, do not have any recognizable pattern determining their secretion by exosomes. Under this scenario, machine learning techniques provide a suitable approach to predict proteins secreted by exosomes. In this work, we specifically used RF, as they have been shown to exhibit high prediction accuracy in many biological problems and with different types of data [24]. Moreover, RF are more intuitive and have less parameters that need optimization than other machine learning algorithms such as support vector machines, which are often applied in classification [25].

**Fig. 2:**
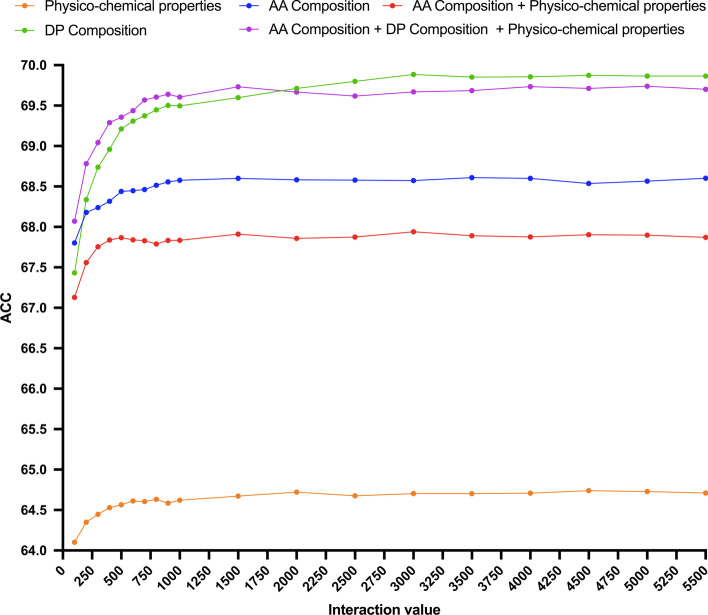
Performance of esRF models predicting proteins secreted by exosomes. Figure shows the accuracy (ACC, Y-axis) in percentage of esRF models trained on exosome training datasets with sequence features consisting of physico-chemical properties (orange), amino acid (AA) composition (blue), combination of amino acid composition and physico-chemical properties (red), dipeptide composition (DP) (green) and combination of all of them (purple) at increasing interaction values (X-axis). Accuracy was obtained under tenfold-cross validation experiments (standard deviations not shown) that were repeated 10 times

To generate prediction models, we trained and evaluated various RF under tenfold cross-validation classification experiments (details in Methods) in the described training dataset translated into feature vectors consisting of amino acid composition, physico-chemical properties, dipeptide composition, the combination of amino acid composition plus physico-chemical properties and the combination of all of them (details in Methods). We chose these features since they can be obtained from the sequences alone. Hereinafter, we will refer to the RF models generated to predict protein secretion by exosomes as esRF. As shown in Fig. 2, in all sequence feature vectors the accuracy of esRF improved as the interaction value increased, stabilizing approximately at a value of 1500. An esRF model trained on dipeptide composition with an interaction value of 3000 reached the top classification accuracy, 69.88% ± 2.08. This esRF model produced classifications with AUC of 0.76 ± 0.03 and MCC of 0.40 ± 0.05 (Table 1). Note that training esRF models on additional features to dipeptide composition, such as physico-chemical properties and amino acid composition, did not improve the accuracy of the predictions.

**Table 1:** Performance of top esRF models generated in this study

	Dataset translation				
	PCP	AA	PCP + AA	DP	PCP + AA + DP
I	4500	3500	3000	3000	5000
ACC (%)	64.74 ± 1.94	68.61 ± 2.05	67.94 ± 2.02	69.88 ± 2.08	69.74 ± 1.93
AUC	0.69 ± 0.04	0.75 ± 0.03	0.72 ± 0.06	0.76 ± 0.03	0.76 ± 0.04
MCC	0.29 ± 0.06	0.37 ± 0.04	0.36 ± 0.05	0.40 ± 0.05	0.39 ± 0.05

Table reports the accuracy (ACC), area under the curve (AUC) and Matthews correlation coefficient (MCC) of the top performing esRF models obtained with the indicated interaction value (I) by training in physico-chemical properties (PCP), amino acid composition (AA), combination of physico-chemical properties and amino acid composition (PCP + AA), dipeptide composition (DP) and combination of physico-chemical properties, amino acid composition and dipeptide composition (PCP + AA + DP). Performance values were obtained under 10-cross validation experiments that were repeated 10 times

The features used for training have a major impact in the performance of the models and we cannot discard the potential benefit of having considered more complex and/or additional features like gene ontology (GO) [26], evolutionary information [27, 28] and/or protein profile-alignments [29]. The inclusion of evolutionary and profile-alignment features is an ingenious manner to enhance datasets and it is of particularly interest when instances for training are limited. However, the use of these types of features, in particular those based on profile-alignments, can cloud former efforts to decrease sequence similarity in the training datasets and demands much computational power. The use of knowledge-based annotations such as GO terms has shown promising results in protein classification problems [26, 30]. However, these annotations are not readily available for all the proteins and will need to be predicted or skipped, thus hampering the utility of the resulting models. Combining different features like those mentioned above could also lead to gains in performance but model overfitting becomes a concern when training in numerous features [31]. Moreover, combining complex features is not trivial and can impact the performance of the models [32, 33]. Therefore, in this work we trained and evaluated models on simple and few sequence-features that could be easily extracted and combined.

To further assess the predictive power of top esRF models selected in cross-validation, we tested them in an independent test dataset consisting of 2346 exosome proteins and 3443 non-exosome proteins generated as indicated in Methods. The similarity between the training and the independent test dataset was very low; overall the average sequence identity between the two datasets was of 12.01 ± 3.30 (%). More measures of identity between these two datasets are provided in Additional file 1: Figure S1. For testing, the independent dataset was translated into feature vectors matching the relevant esRF and their performance is shown in Table 2. All esRF models were able to discriminate proteins secreted by exosomes better than in cross-validation exhibiting an ACC over 68%. The largest ACC was reached again by the esRF model trained on dipeptide composition (ACC = 75.73%). The fact that the performance of esRF models in the independent test dataset was better than in cross-validation along with the larger number of sequences used for model building sharing little sequence similarity supports the robustness of our esRF models to predict proteins secreted by exosomes.

**Table 2:** Performance of esRF models in an independent testing dataset

	Dataset translation				
	PCP	AA	PCP + AA	DP	PCP + AA + DP
ACC (%)	68.99	71.49	71.41	75.73	75.54
AUC	0.765	0.793	0.795	0.840	0.839
MCC	0.378	0.424	0.422	0.505	0.503

Table reports the accuracy (ACC %), area under the curve (AUC) and Matthews correlation coefficient (MCC) reached in the independent dataset by the top performing esRF model obtained by training in sequence features consisting of physico-chemical properties (PCP), amino acid composition (AA), combination of global properties and amino acid composition (PCP + AA), dipeptide composition (DP) and all of them (PCP + AA + DP)

### Comparison of esRF with related methods

Currently, there is not any specific tool to predict protein secretion by exosomes. However, there are a few bioinformatics tools aimed to predict proteins secreted by unconventional pathways [16], and among them, we selected SecretomeP [17] and OutCyte [21], which are both available for free public use over the internet, for comparison with our method. SecretomeP is based on neural networks trained on a dataset of 3654 mammalian proteins that are secreted by unconventional pathways, including exosome cargo proteins [17], and it is often considered as a reference tool. OutCyte is a novel tool related with SecretomeP, also based on neural networks, but yielding contrasting predictions when tested in the human proteome [21].

We evaluated the SE, SP and ACC of SecretomeP and OutCyte in our independent dataset and compared the results with those obtained by our top esRF model trained on dipeptide composition. As shown in Table 3, our esRF model produced values of SE and SP of 0.73 and 0.78 respectively, both clearly superior to those obtained with SecretomeP and OutCyte. The ACC of our esRF model in the test dataset was also clearly superior to that of SecretomeP and OutCyte (75.73% vs 45.14% and 54.39%, respectively). Overall, these results indicate that SecretomeP and OutCyte are surprisingly unable to predict proteins secreted by exosomes, which highlights the utility of the esRF models developed here.

**Table 3:** Comparative performance of Outcyte, SecretomeP and esRF

	Prediction method		
	OutCyte	SecretomeP	esRF
SE	0.32	0.25	0.73
SP	0.72	0.65	0.78
ACC (%)	54.39	45.14	75.73

SecretomeP, OutCyte and esRF models were evaluated in our independent dataset and compared with regard to sensitivity (SE), specificity (SP) and accuracy (ACC). SecretomeP predictions were obtained at http://www.cbs.dtu.dk/services/SecretomeP/ selecting the mammalian option and the default prediction threshold of 0.6. OutCyte predictions were obtained at http://www.outcyte.com selecting the “OutCyte-UPS” model and default settings

It is worth nothing that exosome secretion of proteins could have been approached as a sub-cellular location problem. However, the fact that proteins in exosomes can also have different locations complicates this approach. A suitable solution, already applied to predict sub-cellular location of proteins, would be to train classification models considering multi-labels [26, 29, 30]. However, to our knowledge, not even the most recent methods of sub-cellular location prediction consider exosomes within their predicted locations [26–30, 32–34]. Therefore, we suggest combining our models of exosome secretion with those that can predict sub-cellular location.

### ExoPred web server

**Fig. 3:**
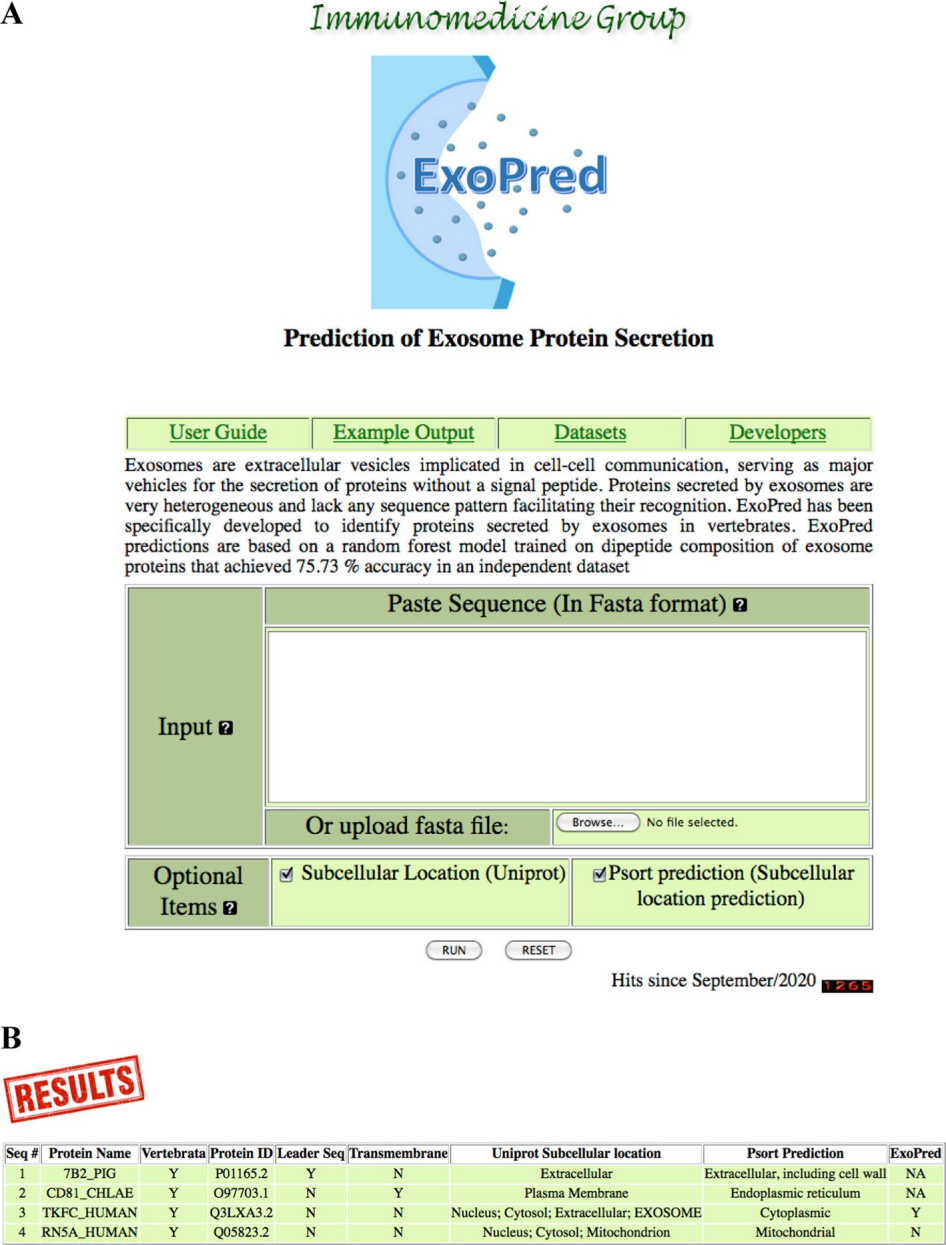
ExoPred web server. **a** ExoPred interface. **b** ExoPred result page with sub-cellular and PSORT predictions selected. The information shown is the following (from left to right): Number of the sequence; name of the sequence in the FASTA file; belonging to vertebrates (Y/N); UNIPROT ID; presence of predicted signal peptides (Y/N) or transmembrane regions (Y/N); sub-cellular location annotated in UNIPROT; sub-cellular location predicted by PSORT [35]; and, finally, exosome secretion prediction (Y/N)

Given the results described above and the relevance of predicting proteins secreted by exosomes, we developed a web-based tool, ExoPred, which implements our top esRF for free public use (http://imath.med.ucm.es/exopred/). The ExoPred interface, shown in Fig. 3a, has been designed for simple and intuitive use. The input data for ExoPred can be one or several protein sequences in FASTA format, which can be pasted or uploaded to the server. In ExoPred, users can also select to retrieve the sub-cellular location of input proteins as annotated in UNIPROT and/or predict such sub-cellular location using PSORT (version II) [35]. After submission, ExoPred first runs a BLASTP [36] against the UNIPROT database and processes the BLAST output to identify the UNIPROT identifier (ID) of protein hits with identity higher than 90% and over 90% of their entire length. After these identifiers, ExoPred will then retrieve taxa and sub-cellular location information from UNIPROT annotations and transfer it to the relevant input query proteins. ExoPred will also detect those proteins with leader sequences and transmembrane regions using SignalP [37] and TMHMM [38] and predict sub-cellular locations using PSORT [35].

The model for predicting exosome secretion is only executed in proteins from vertebrate and without a signal peptide or transmembrane regions. ExoPred output consists of a table reporting by default whether input proteins are from vertebrate (Y/N), contain a signal peptide (Y/N) or transmembrane regions (Y/N) and can be secreted via exosomes (Y/N). As show in Fig. 3b, ExoPred will also show the sub-cellular location of input proteins annotated in UNIPROT and predicted by PSORT if the relevant options were checked at submission. Exosome secretion predictions will show as NA (not available) for input proteins that do not meet the criteria mentioned above. For proteins without UNIPROT equivalents, ExoPred will still determine whether they can be secreted by exosomes as long as they have no predicted signal peptide or transmembrane regions. In these cases, the field taxa, and UNIPROT sub-cellular-location, when selected, will show as not found.

## Conclusions

Exosomes have a relevant role in intercellular communication in eukaryotes, representing a major vehicle to secret leaderless proteins. Currently, no method is available to specifically predict protein secretion by exosomes. Here, we developed ExoPred, a web-based tool to predict proteins secreted by exosomes. ExoPred predictions are based on random forests models that achieved 75.73% accuracy in an independent dataset. Predicting and annotating that a particular protein can be secreted by exosomes is clearly relevant, as it is indicative of a potential role in cell communication and suggests new untapped functions. Therefore, we plan to release a standalone version for resources and bioinformaticians providing protein sequence annotations.

## Methods

### Generation of exosome protein datasets

In this study, we assembled two non-overlapping protein datasets, a training dataset and an independent test dataset, both including exosome and non-exosome proteins. Exosome proteins in the training dataset where obtained from QuickGo [39] after the GO term “extracellular exosome” ([GO: 0070062]) and from ExoCarta database [40]. Non-vertebrate proteins and ExoCarta proteins without UNIPROT [41] representation were not considered as well as unreviewed proteins and those with a global annotation score lower than 3 out of 5 as annotated by UNIPROT. Likewise, exosome proteins including signal peptides and/or transmembrane regions were discarded. CD-HIT software [42] was applied to reduce sequence similarity so that exosome proteins in the training dataset do not share more than 80% identity. Non-exosome proteins in the training dataset were randomly collected from UNIPROT and obeyed to the same criteria than exosome proteins (reviewed, from vertebrate, global annotation ≥ 3, sequence similarity under 80% and exclusion of proteins with a leader sequence and/or transmembrane regions). Exosome proteins in the test dataset met the same criteria than those in the training dataset but were obtained from ExoCarta without considering UNIPROT quality annotations. Non-exosome proteins in the test dataset were also obtained as described earlier. Datasets are available at http://imath.med.ucm.es/exopred/datasets/.

### Sequence similarity analyses

Sequence similarity in training and independent datasets was analyzed after pairwise sequence alignments generated using the Needleman-Wunsch global alignment algorithm implemented by the *needle* application of the EMBOSS package [43]. To obtain a measure of sequence similarity in a dataset, all sequences were aligned pairwise but with themselves (for a dataset with *N* sequences there will be *N* x *N-1* alignments), identities were obtained for each alignment and the average identity was computed.

### Model building and evaluation

Models to predict proteins secreted by exosomes were built using the Waikato Environment for Knowledge Analysis (WEKA) package [44]. WEKA provides a framework for data classification, clustering and feature selection using a large collection of machine learning algorithms. In WEKA, exosome protein secretion models were trained and evaluated under the application EXPLORER, using RF as classification algorithms. Classification with RF operate by applying multiple decision trees generated during training and outputting a modal decision [45, 46]. Different RF models were obtained by varying the interaction value of the algorithm (100, 150, 200, 250, 300, 350, 400, 450, 500, 550, 600, 650, 700, 750, 800, 850, 900, 950, 1000, 1500, 2000, 2500, 3000, 3500, 4000, 4500, 5000, 5500, 6000, 8000, 10,000). The interaction value indicates the number of trees in RF. As input for WEKA, we used five distinct training datasets in ARFF format resulting of translating the amino acid sequences in the exosome training dataset into feature vectors of equal length. Sequence feature vectors consisted of amino acid composition, physico-chemical properties, dipeptide composition, the combination of amino acid composition and physico-chemical properties, and the combination of all of them. Amino acid and dipeptide compositions of protein sequences were computed as described elsewhere [47]. The amino acid composition feature vector contains 20 values indicating the proportion of each of the 20 natural amino acids in the sequence. The dipeptide composition feature vector contains 400 values depicting the proportion of all possible pair of amino acids (20 × 20) in the sequences. The physico-chemical property feature vector contains 11 values, *P*_*i*_*,* computed for each sequence after 11 distinct amino acid properties. For each physico-chemical property, *i*, *P*_*i*_, was computed using Eq. 1 where *p*_*i*_*a*_n_ is the relevant normalized physico-chemical property of amino acid, *a*, at the *n* position of a given protein sequence, and *N* is the total number of amino acid residues in the sequence.

1 $$P_{i} = \frac{{\sum\nolimits_{n = 1}^{N} {p_{i} a_{n} } }}{N}$$

The 11 amino acid physico-chemical properties used in this study included average flexibility indices [48], residue volume [49], relative mutability [50], net charge [51], optimized side chain interaction parameter [52], polarity [53], alpha-helix propensity derived from designed sequences [54], beta-sheet propensity derived from designed sequences [54], amphiphilicity index [55], modified Kyte-Doolittle hydrophobicity scale [56] and aromaticity. Combination feature vectors were obtained by merging the relevant vectors. As a result, the amino acid composition and physico-chemical property feature vector contains 33 values resulting of merging 20 amino acid composition values and 11 physico-chemical property values per sequence into a single vector. Likewise, the combined feature vector containing amino acid and dipeptide compositions, and physico-chemical properties contains 433 values.

RF models were trained and evaluated in tenfold cross-validation classification experiments that were repeated 10 times. Best performing models that were obtained by training in the noted sequence features were also evaluated in the test dataset.

### Measures of performance

The performance of RF models was obtained by computing threshold-dependent measures such as sensitivity (SE), specificity (SP), Matthews correlation coefficient (MCC) and accuracy (ACC) using Eqs. 2, 3, 4 and 5, respectively. These measurements are expressed in terms of true positive (TP), false negative (FN), true negative (TN) and false positive (FP) predictions.

2 $$SE = \frac{TP}{{TP + FN}}$$

3 $$SP = \frac{TN}{{TN + FP}}$$

4 $$MCC = \frac{{\left( {TP \times TN} \right) - \left( {FN \times FN} \right)}}{{\sqrt {\left( {TN + FN} \right)\left( {TP + FN} \right)\left( {TN + FP} \right)\left( {TP + FP} \right)} }}$$

5 $$ACC = \frac{{\left( {TP + TN} \right)}}{{\left( {TP + FP + TN + FN} \right)}} \times 100$$

The performance of RF models was also evaluated by computing the area under the curve (AUC) resulting from plotting SE *vs* 1—SP at different thresholds. An AUC value of 0.5 corresponds to a random prediction, while a value of 1 reflects a perfect prediction.

### Prediction of unconventional protein secretion using SecretomeP and OutCyte

SecretomeP [17] is a web-based tool for predicting unconventional protein secretion available at http://www.cbs.dtu.dk/services/SecretomeP/. For comparative analysis, SecretomeP was used to predict exosome and non-exosome proteins in the test dataset selecting the “Mammalian” option. Proteins with “NN-scores” higher than 0.6 were considered as secreted by an unconventional pathway, as indicated in the web page. OutCyte [21] is another web-tool, available at http://www.outcyte.com, for the prediction of unconventional protein secretion based on convolutional neural networks. For comparative analysis, proteins included in the test dataset were subjected to OutCyte predictions, using the model “OutCyte-UPS” with the default settings.

### Web implementation

Exosome prediction models were implemented for free public use on the Web using a Python CGI (Common Gateway Interface) script that executes the predictions on user-provided input data and returns the results to the browser. The front-end web interface was developed using Hyper Text Markup Language (HTML) in combination with Cascading Style Sheets (CSS) and JavaScript. Web page administration is done using Apache HTTP Server (https://httpd.apache.org).

## Supplementary Information

**Additional file 1**:** Figure S1**. Sequence similarity in training and testing datasets. The figure shows the percentage of identity between exosome proteins, non-exosome proteins, exosome vs non-exosome proteins and all sequences found in the training datasets and independent test datasets. It also shows the percentage of identity between proteins of the training dataset with those of the independent test dataset considering exosome proteins, non-exosome proteins, exosome proteins vs non-exosome proteins and all proteins. Sequence identity was computed as indicated in Methods and reported as an average identity with their standard deviation.

## Data Availability

Authors confirm that all relevant data are included in the article.

## Abbreviations

AA: Amino acid composition
ACC: Accuracy
AUC: Area under the curve
DP: Dipeptide composition
ER: Endoplasmic reticulum
GA: Golgi aparatus
MCC: Mathew’s correlation coefficient
PCP: Physico-chemical properties
RF: Random forests
SE: Sensitivity
SP: Specificity
