# Supplementary figures and images for: Prediction of unconventional protein secretion by exosomes
Source: BMC Bioinformatics. 2021 Jun 16;22:333. doi: 10.1186/s12859-021-04219-z (PMC8210391; doi:10.1186/s12859-021-04219-z)

- Exosome vs Exosome
- Non-exosome vs Non-exosome
- Exosome vs Non-exosome
- All vs All

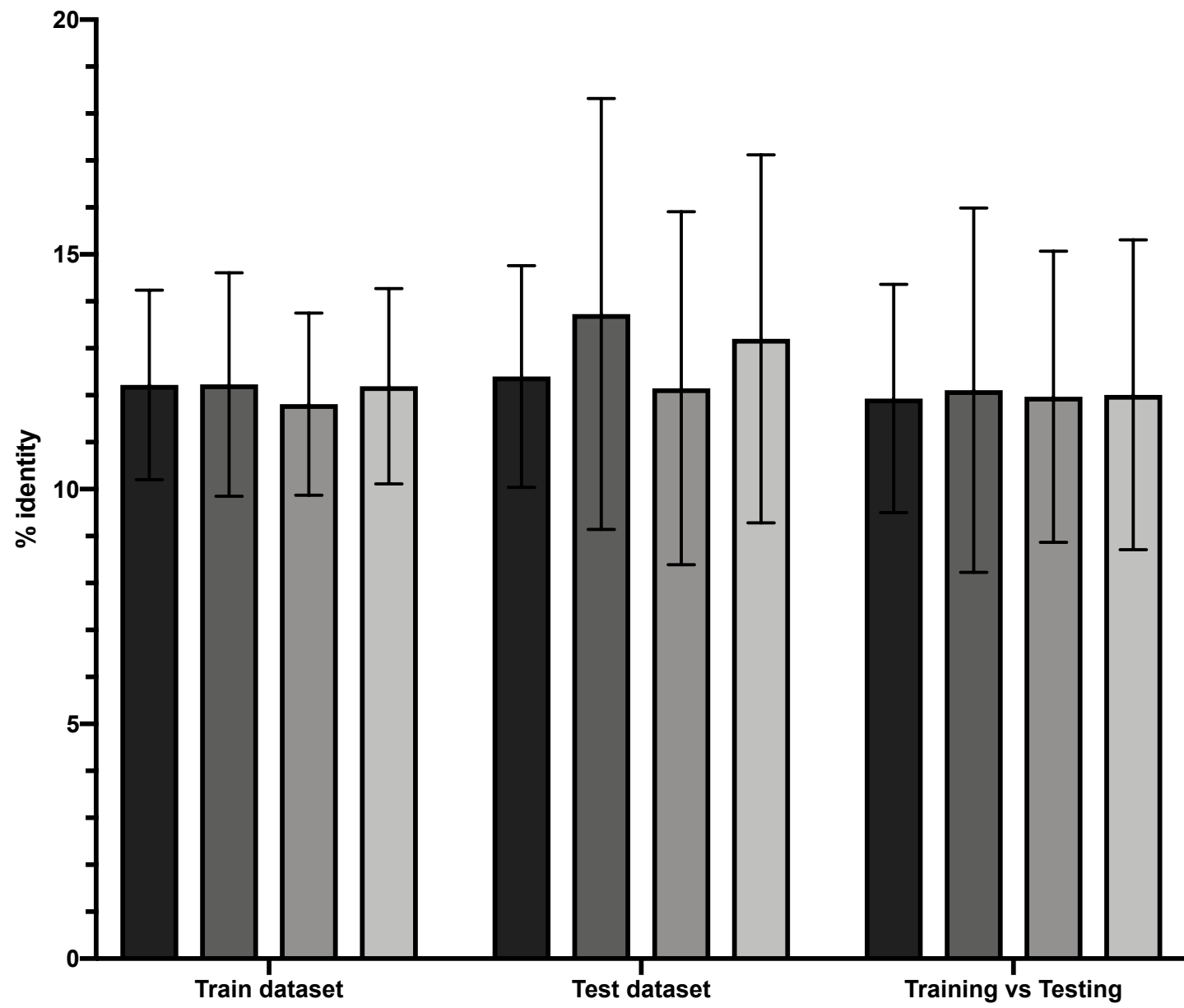

Supplement: Supplementary file 1 — Additional file 1: Figure S1. Sequence similarity in training and testing datasets. The figure shows the percentage of identity between exosome proteins, non-exosome proteins, exosome vs non-exosome proteins and all sequences found in the training datasets and independent test datasets. It also shows the percentage of identity between proteins of the training dataset with those of the independent test dataset considering exosome proteins, non-exosome proteins, exosome proteins vs non-exosome proteins and all proteins. Sequence identity was computed as indicated in Methods and reported as an average identity with their standard deviation. [file 12859_2021_4219_MOESM1_ESM.pdf]
